# My journey from an early researcher to an aspiring medic: A dance from bench to bedside

**DOI:** 10.1113/EP092239

**Published:** 2024-11-27

**Authors:** Jaspreet Kaur

**Affiliations:** ^1^ School of Medicine and Dentistry University of Central Lancashire Preston UK

1

Whilst working as a health‐care assistant in a dementia care home, I remember being exposed to the tremendous impact that diseases such as Parkinson's and Alzheimer's wreak upon an individual's life. I vividly remember one of the patients, a French model, who could recall only her signature hand wave, whereas another patient with a PhD had a vocabulary that was reduced to only a couple of coding words. These individuals ignited my inquisitive nature, which is why I pursued a Bachelor's in Biomedical Science at the University of Plymouth in Devon, UK to begin my understanding of the complexities of a human body malfunctioning.

My navigation throughout my undergraduate degree allowed me to develop a strong understanding of the delicate structure and functions within the nervous system. Throughout my undergraduate degree, I was able to hone my clinical skills through my laboratory time. Especially during my summer internship, I carried out various lung function tests in order to obtain an in‐depth understanding of the likely prognosis. Realizing that everyone has different lung capacities and how different diseases, such as chronic obstructive pulmonary disease and asthma, affect individuals captured my interest in obtaining a deeper understanding of the impact on their daily lives. This was the beginning of my commitment to physiology. Hence, my curiosity fuelled my interest in exploring how hypoxia affects physiological processes, such as the way in which hypoxia activates peripheral chemoreceptors in the sympathetic nervous system (Prabhakar et al., 2015), which could be assessed via heart rate variability. This was a very interesting concept that I wanted to explore. Consequently, I was able to secure a summer internship focusing on ‘The effect of breathing hypoxic gas (15% FIO_2_) on physiological and behavioral outcomes during simulated driving in healthy subjects’ (Kaur, Thynne, et al., 2024). We found that breathing rate showed a pattern of decrease with both medical air and hypoxia; the majority of the findings were not significant. Doing this research showed me the importance of reporting negative findings. These results potentially reassure policymakers that the chances of road safety being decreased are minimal when allowing a mildly hypoxic person to drive.

Following my summer internship, I secured a master's thesis project on ‘An investigation of the acute effects of dance on heart rate variability in people with Parkinson's (PwP)’. This allowed me to combine my new skill sets of LabChart analysis, critical and statistical analysis, which aided me in publishing a primary research paper with my initial interest in understanding the difficulties of neurodegenerative disease (Kaur, Thynne et al., 2024). Having to develop a research protocol, a detailed method section and getting ethics approved, reasoning every step was as fun as overwhelming at times. Along with uncertainty, at times there were technical and logistical issues, sometimes not involving the research itself, but factors outside our control. Getting to the Bournemouth venue for the dance academy on the train proved to be an endeavour when we almost missed our stop because our carriage did not meet the platform owing to the train being too long. Taking the train also involved three difficult changes, and after our close call, we eventually decided to stick with the reliability of a car, although we unfortunately encountered a fair amount of traffic. However, the traffic gave us a great amount of time to enjoy the baked goods made by the wife of my supervisor. My supervisor and I were incredibly chatty in the car, usually talking about our family and friends. Alongside the heartfelt talks, the banana bread, oat cookies, flap jacks and other homemade goods kept us going during the long journeys. Likewise, getting used to unfamiliar equipment lengthened the research process. However, overcoming these setbacks showed me that there is always a redirection to a better outcome.

I was lucky to have a supportive supervisor who guided me over every hurdle and educated me on how to approach the next steps. In fact, thanks to my unending stubbornness and my supervisor's patience, the hard experiences of travelling from Plymouth to Bournemouth were made manageable; likewise, making sure that all the devices are synchronized to the laptop via Bluetooth and the uncertainty of whether we will have participants the following week. This meant that we always had to have a reserved list of participants and early confirmation. This taught me the importance of time management and always to have a ‘plan B’ in place. The long hours of analysing data, although monotonous, did not limit the quality of the manuscript drafts. Furthermore, learning how to use a new piece of equipment (in this case, the Equivital vest), trying to connect the sensor electronic model (SEM) device to the vest (Figure 1) and then connecting the whole system to the software on the laptop was initially challenging, but eventually became satisfying. The added challenge of collecting data from two participants at the same time on the SPSS (Statistical Package for Social Sciences) sheet required me to work in a systematic order and under pressure, to make sure the right participant's information was recorded and to be wary of any glitches. Our technique of collecting data from two participants simultaneously was something even the manufacturers were unsure of, but it worked successfully. There were many trials and errors, and the best way to try out the new equipment was by dancing in the campus corridors, trying to simulate the same environment and distance to see whether the Bluetooth would work. This part of the preparation was like the cherry on the cake, and it felt amazing, knowing that everything was working exactly how we wanted it to.

**FIGURE 1:**
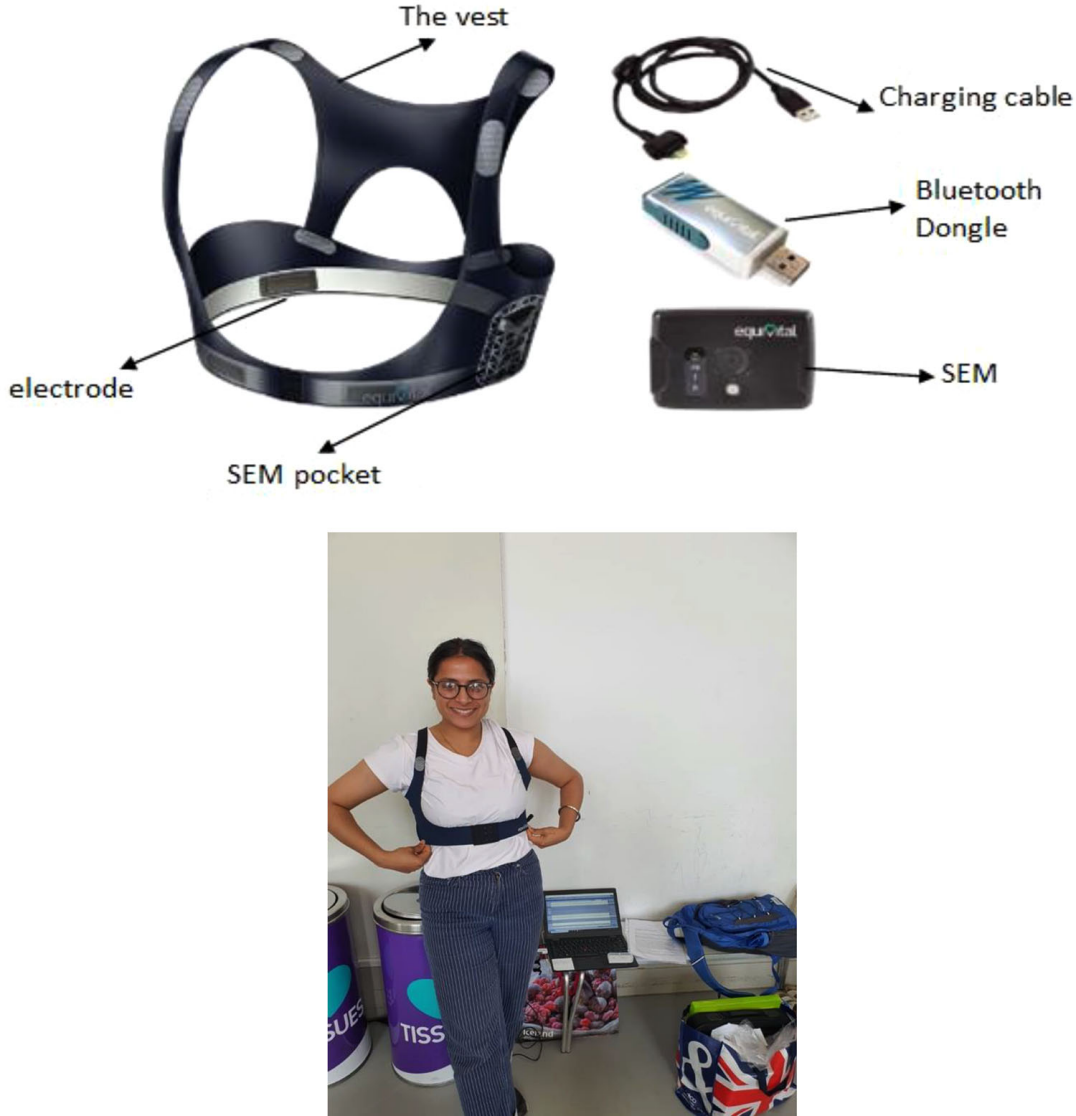
The Equivital vest and the equipment that was used to collect data, and the author wearing it. Abbreviation: SEM, sensor electronic model.

These challenges helped me to grow my resilience, and I learned to tackle any issues that came my way through intuition and consultation. However, despite the challenges, the journey itself is the most rewarding part of my research career. The beauty of finding answers that highlight the gaps in the research field that my projects potentially answer is the most fulfilling feeling! The Dance Parkinson's research taught me how to work in an interprofessional environment, because this project involved a collaboration with members from the physiotherapy and music departments. Having a diverse group with various skill sets gave me a strong base to start this research. For example, the choreographed dance developed by dance teachers was adapted to the needs of PwP. The collaboration of different departments facilitated the effort by multi‐disciplinary team to bring PwP together. There were definitely challenges while collecting data, especially because of PwP, who are vulnerable and need support while putting on a vest (Figure 1). The vest has electrodes that have to be damp and need to touch the skin inorder to collect the data. Directly touching the skin can be very daunting, and the vest can be uncomfortable to wear for a long period of time. Another issue that we faced was the differences in structural anatomy of participants, and collecting data from multiple participants was challenging because we only had a few vests of specific sizes.

I have watched how all our hard work came to fruition, especially when both of our final projects were presented at The Physiological Society conferences. The hypoxia study was presented as a poster presentation at the Annual Physiology 2023 Conference, in Harrogate, UK. This was my first poster presentation at a conference (Kaur et al., 2023), hence being nervous would be an understatement; I was very frightened. However, my supervisor was really kind and guided me throughout the whole process and let me take over as soon as I got the hang of it. The challenging part was when I was asked questions. Although I was able to answer the majority of the questions by myself, there were times when tough questions were asked by the scientists, which required more thinking ‘out of the box’. My Parkinson's study was presented at the Annual Physiology in Focus 2024 Conference, Newcastle, UK. Given that this was my second poster presentation (Kaur, Hulbert et al., 2024), I was a bit more confident, in the sense I had an idea of what to expect, but nervous about the type of questions I would be asked. Additionally, the feedback was very positive, and the scientists helped me to analyse my own data critically. For example, I was asked whether I analysed data comparing PwP who have danced for >6 months compared with those who were beginners. Although this statistical analysis was not done by me, it was definitely a valid point, and this type of data would have been interesting to analyse. Last but not least, my hypoxia research was published in Physiological Reports in 2024 (Kaur, Thynne et al., 2024). A take‐home message from my experiences as an early career researcher is that it is not an easy path, and it is not always meant to be. Doing research requires time, a lot of thought input and many setbacks that might even lead to an entire remoulding of the initial research idea.

My research so far has been highly motivational, because I have collected data from a younger healthy population during my hypoxia research project, in addition to seeing PwP dancing to personalized choreography. Having covered all age groups shows how important research is for everyone's health, no matter the age, from understanding the physiological effects of hypoxia while driving and looking into the safety of road users to looking at how holistical therapy, such as dance, is beneficial for the older PwP population. My studies have shown the impact on quality of life across age groups. This was seen when I spoke to the participants after their dance and asked them how they felt, to which they replied, saying, ‘I look forward to dancing every week’, and ‘dancing makes me happy’. Hearing their feedback made me feel grateful and privileged that I was able to be part of their lives. Collecting their data to show how effective dance is for their health was also very reassuring for them. Moreover, attending these dance sessions was a very much an eye opener, because I realized that although everyone had Parkinson's, everyone had very different presentation of it, hence it also made me realize the importance of a personalized management plan for each individual patient. Although cellular research [e.g., for Parkinson's disease, focusing on developing therapies that activate the REarranged during Transfection (RET) receptor pathway and restore the function of dopaminergic neurons in PwP] is important, I strongly believe that clinical research with human participants is equally essential. From my own research, I have seen how clinical research gives a better idea of the effects on living people in the present moment and improves quality of life. I like to say that if holistic approaches, such as dance, were given as a ‘medication tablet’, I am sure many would be happy to take it.

After completing my Master's in Biomedical Science (Clinical Neuroscience) at the University of Plymouth in Devon, UK, I have become an aspiring medical student at the University of Central Lancashire in Preston, UK. I now realize how important it is to combine my role as a biomedical scientist early career researcher with the knowledge I will gain through my medical education of the complexities of the human body, and personalized medicine will allow me to give the best possible care to my patients. Furthermore, I now have a better understanding of how to conduct research to enable me to apply my skill set and knowledge to clinical research in medicine and become an academic clinician.

The most challenging endeavour for me so far has been to juggle research while pursuing my medical degree. However, it has been one of the most rewarding aspects of my education. My experience to date has not only strengthened my commitment to both fields but has also nurtured personal and professional growth. From going to lectures in medical school to using my breaks in between the lectures to write up a paper over Zoom with my supervisor and a particularly intelligent, proofreading friend of mine, not only did I learn how to manage time, but I also made sure to not get burnt out, by making sure that I made time for running and badminton, which helped me to stay sane. The advantage of engaging in research alongside medical studies has allowed me to apply scientific principles of how dance therapy benefits the parasympathetic system and gait in PwP. This also links to clinical scenarios, such as informing the elderly population, and especially PwP, about the benefits of exercise as a regular form of fitness. This allows me to reference my own research, giving people reassurance, because I have witnessed the positive impact of dance through first‐hand experience. Hence, my research on Parkinson's disease has deepened my understanding of the physiological impact that dance can have on acute and long‐term effects on PwP, and how it can be part of their personalized care plan. Having seen the world from both ends, it is fair for me to conclude that a deeper understanding of physiology and medicine comes not only from clinical practice but also from contributing to scientific knowledge that drives medical advancements.

## AUTHOR CONTRIBUTIONS

Sole author.

## CONFLICT OF INTEREST

None declared.

## FUNDING INFORMATION

Funding was provided by the government through student finance.
